# Quaternion to Euler angles conversion: A direct, general and computationally efficient method

**DOI:** 10.1371/journal.pone.0276302

**Published:** 2022-11-10

**Authors:** Evandro Bernardes, Stéphane Viollet

**Affiliations:** Aix-Marseille Université, CNRS, ISM, Marseille, France; Ningbo University, CHINA

## Abstract

Current methods of the conversion between a rotation quaternion and Euler angles are either a complicated set of multiple sequence-specific implementations, or a complicated method relying on multiple matrix multiplications. In this paper a general formula is presented for extracting the Euler angles in any desired sequence from a unit quaternion. This is a direct method, in that no intermediate conversion step is required (no quaternion-to-rotation matrix conversion, for example) and it is general because it works with all 12 possible sequences of rotations. A closed formula was first developed for extracting angles in any of the 12 possible sequences, both “Proper Euler angles” and “Tait-Bryan angles”. The resulting algorithm was compared with a popular implementation of the matrix-to-Euler angle algorithm, which involves a quaternion-to-matrix conversion in the first computational step. Lastly, a single-page pseudo-code implementation of this algorithm is presented, illustrating its conciseness and straightforward implementation. With an execution speed 30 times faster than the classical method, our algorithm can be of great interest in every aspect.

## 1 Introduction

When dealing with 3D orientation problems, many different formalisms can be used to describe a given rotation [1], each of which has its own set of advantages and shortcomings. Arguably the most direct representation of a 3D rotation is a matrix *R* ∈ *SO*(3), where *SO*(3) is the group of invertible 3 × 3 matrices such that det(*R*) = 1 and $RR^{T}=R^{T}R=I$, where I is the identity matrix. These rotation matrices represent direct linear transformations such that, with $v\in R^{3}$:

$$\begin{array}{c} v_{\text{rotated}}=R\;v \end{array}$$ (1)

Apart from being simple to use, a rotation matrix also has the advantage of being continuous, and a simple matrix multiplication can be used to compose rotations: *R* = *R*_2_*R*_1_ is the rotation matrix corresponding to a rotation by *R*_1_ followed by a rotation by *R*_2_. 3D rotation matrices have some numerical shortcomings, however. For example, as many as 9 numbers (and 6 constraints) are required to represent a 3 degree of freedom rotation, and it can be difficult and computationally costly to orthogonalize a rotation matrix numerically [2] (i.e., to check that the matrix has its determinant equal to 1 and its inverse equal to its transpose, which is necessary to compensate for the accumulated floating point errors).

However, it is possible to parametrize this rotation matrix with a smaller set of numbers [3]. One of the most usual set of parameters are the Euler angles. The approach consists in decomposing the 3D rotation matrix into the product of three rotations:

$$\begin{array}{c} R=R_{\theta_{3}\;e_{3}}\;R_{\theta_{2}\;e_{2}}\;R_{\theta_{1}\;e_{1}} \end{array}$$ (2)

Where *R*_*θ* ***e***_ is a rotation by the angle *θ* around the axis ***e***, and the consecutive axes are orthogonal (***e***_1_ ⋅ ***e***_2_ = ***e***_2_ ⋅ ***e***_3_ = 0). The advantages of using Euler angles include the fact that only three numbers have to be stored, and due to their familiarity, they can be more easily understood, which explains why they are still being so widely used, even in cases where other forms of representation may be more appropriate. The use of Euler angles also has several disadvantages. For example, they are discontinuous and it is difficult to directly compose two 3D rotations expressed in Euler angles. Euler angles are also affected by the phenomenon commonly called “gymbal lock”: when two axes become aligned, making the system underdetermined, special care has to be taken. In addition, since there are 12 possible axis sequences (24, when considering the difference between “intrinsic” and “extrinsic” rotations), the correct sequence has to be checked in the case of each application. An arguably preferable parametrization are quaternions. A quaternion is a hypercomplex number defined by one real part and three distinct imaginary parts (which can also be regarded as the vector part). When the norm of a quaternion is equal to 1, quaternions are a useful and efficient representation of 3D orientation: they can be composed as easily as rotation matrices, they are continuous, and they are easily constructed from the axis-angle representation. In addition, quaternions can be normalized trivially, which is much more efficient than having to cope with the corresponding matrix orthogonalization problem. For these reasons, most 3D graphical applications and rotation engines carry quaternions under the hood. Besides these advantages, Euler angles are still being preferred by many authors: Euler angles are the most familiar concept to most engineers and researchers. In addition, in the case of many problems in which there exists only one degree of freedom, angles can suffice.

To be able to perform fast calculations with quaternions and at the same time analyze rotations using angles, it might be necessary to have an efficient method of converting the one set of parameters to the other. Calculating the corresponding quaternion (or rotation matrix) for a given set of Euler angles is trivial. Extracting the Euler angles is much harder, however. One of the following two methods has generally been used up to now. The first method consists in adopting a different set of formulas for each possible angle sequence [4], which is difficult to implement and debug. The second method is that described in [5]. SciPy [6], for example, a widely used scientific library for the Python programming language, implements this method. It converts rotation matrices into Euler angles and involves many different matrix multiplications, including the inverse trigonometric functions required, which are naturally computationally costly. In addition, if rotations are stored in the form of quaternions (as is usually the case in many of the 3D rendering software tools dealing with rotations), an additional conversion step from quaternions to rotation matrices is necessary.

Since many robotic, graphic and other high-level applications involve the use of quaternions (even if they are hidden from the user), it can be necessary to have a concise, efficient method for the conversion between quaternions and Euler angles. The direct conversion formula from quaternions to Euler angles presented here requires fewer computational steps and less expensive computational resources. Moreover, this conversion formula is much simpler to implement and debug, making it a great option for any new applications needing to implement this kind of conversions.

## 2 Quaternion algebra summary

In this section, the key properties of quaternions are summarized. It is assumed in this work that we are dealing with the classical Hamilton quaternions. Since the definitions concerning quaternion algebra are not perfectly consistent in the literature [7], some of the main notations and definitions used in this study are then presented. Quaternions form a non-commutative division algebra denoted by H, which extends the complex numbers. A quaternion q∈H consists of four components:

$$\begin{array}{c} q=q_{\text{r}}+q_{\text{x}}i+q_{\text{y}}j+q_{\text{z}}k \end{array}$$ (3)

Where $q_{\text{r}},q_{\text{x}},q_{\text{y}},q_{\text{z}}\in R$. All the properties of quaternions can be obtained using its fundamental property, as given by Hamilton:

$$\begin{array}{c} i^{2}=j^{2}=k^{2}=ijk=-1 \end{array}$$ (4)

Using the above properties, the product of two quaternions q and p can be expressed by the Hamilton product:

$$\begin{array}{cc} q\;p & =(p_{r}q_{\text{r}}-p_{x}q_{\text{x}}-p_{y}q_{\text{y}}-p_{z}q_{\text{z}})+(p_{r}q_{\text{x}}+p_{x}q_{\text{r}}-p_{y}q_{\text{z}}+p_{z}q_{\text{y}})i\\ & +(p_{r}q_{\text{y}}+p_{x}q_{\text{z}}+p_{y}q_{\text{r}}-p_{z}q_{\text{x}})j+(p_{r}q_{\text{z}}-p_{x}q_{\text{y}}+p_{y}q_{\text{x}}+p_{z}q_{\text{r}})k \end{array}$$ (5)

For the sake of simplicity, quaternions will be written here as 4 × 1 vectors (with the scalar *q*_*r*_ as the first element):

$$\begin{array}{c} q=[\begin{array}{c} q_{\text{r}}\\ q_{\text{x}}\\ q_{\text{y}}\\ q_{\text{z}} \end{array}]=[\begin{array}{c} q_{\text{r}}\\ q \end{array}] \end{array}$$ (6)

Where $q={\left[\begin{array}{ccc} q_{\text{x}} & q_{\text{y}} & q_{\text{z}} \end{array}\right]}^{T}$ is the the imaginary/vector part of *q*. The Hamilton product between two quaternions in 4-vector form will be denoted by:

$$\begin{array}{c} q⊙p=[\begin{array}{c} q_{\text{r}}\\ q \end{array}]⊙[\begin{array}{c} p_{r}\\ p \end{array}]=[\begin{array}{c} q_{\text{r}}p_{r}-q\cdot p\\ q_{\text{r}}p+p_{r}q+q\times p \end{array}] \end{array}$$ (7)

Defining the conjugate $q^{*}=[\begin{array}{c} q_{\text{r}}\\ -q \end{array}]$ and the absolute value as $\left|q\right|=\sqrt{q_{\text{r}}^{2}+q_{\text{x}}^{2}+q_{\text{y}}^{2}+q_{\text{z}}^{2}}$, the inverse *q*^−1^ of *q* is given by:

$$\begin{array}{c} q^{-1}=\frac{q^{*}}{{\left|q\right|}^{2}} \end{array}$$ (8)

And for any quaternion *q*:

$$\begin{array}{c} q⊙q^{-1}=q^{-1}⊙q=[\begin{array}{c} 1\\ 0 \end{array}] \end{array}$$ (9)

If *q* is a unit quaternion, which means that |*q*| = 1 and *q*^−1^ = q*, it can be used to represent the rotation between two reference frames. Denoting **v**_*A*_ and **v**_*B*_ a vector **v** in frames *A* and *B*, respectively, and $q=q_{A}^{B}$ the unit quaternion corresponding to the rotation from *A* to *B*:

$$\begin{array}{c} {}[\begin{array}{c} 0\\ v_{B} \end{array}]=q_{A}^{B}⊙[\begin{array}{c} 0\\ v_{A} \end{array}]⊙{\left(q_{A}^{B}\right)}^{*} \end{array}$$ (10)

The equivalent rotation matrix is given by:

$$\begin{array}{c} R_{A}^{B}=[\begin{array}{ccc} q_{\text{r}}^{2}+q_{\text{x}}^{2}-q_{\text{y}}^{2}-q_{\text{z}}^{2} & -2q_{\text{r}}q_{\text{z}}+2q_{\text{x}}q_{\text{y}} & 2q_{\text{r}}q_{\text{y}}+2q_{\text{x}}q_{\text{z}}\\ 2q_{\text{r}}q_{\text{z}}+2q_{\text{x}}q_{\text{y}} & q_{\text{r}}^{2}-q_{\text{x}}^{2}+q_{\text{y}}^{2}-q_{\text{z}}^{2} & -2q_{\text{r}}q_{\text{x}}+2q_{\text{y}}q_{\text{z}}\\ -2q_{\text{r}}q_{\text{y}}+2q_{\text{x}}q_{\text{z}} & 2q_{\text{r}}q_{\text{x}}+2q_{\text{y}}q_{\text{z}} & q_{\text{r}}^{2}-q_{\text{x}}^{2}-q_{\text{y}}^{2}+q_{\text{z}}^{2} \end{array}] \end{array}$$ (11)

And the equivalent quaternion for a rotation of an angle *θ* around an axis ***e*** is given by:

$$\begin{array}{c} q_{\theta e}=[\begin{array}{c} \text{cos}(\theta /2)\\ \text{sin}(\theta /2)e \end{array}] \end{array}$$ (12)

## 3 Formula development

In the section, the formula for the conversion between a quaternion and any of the 6 proper Euler angle sequences is derived, and then an adaptation for the 6 remaining Tait-Bryan sequences is demonstrated.

### 3.1 Case 1: Proper Euler angles

Assuming *q* = [*q*_r_, ***q***^*T*^]^*T*^ is unit an known, it can be decomposed as follows:

$$\begin{array}{c} q=[\begin{array}{c} c_{3}\\ s_{3}e \end{array}]⊙[\begin{array}{c} c_{2}\\ s_{2}e^{\prime } \end{array}]⊙[\begin{array}{c} c_{1}\\ s_{1}e \end{array}] \end{array}$$ (13)

In which (for 0 ≤ *θ*_2_ ≤ *π*):

$$\begin{array}{cc} s_{1} & \equiv \text{sin}\left(\theta_{1}/2\right),\;c_{1}\equiv \text{cos}\left(\theta_{1}/2\right)\\ s_{2} & \equiv \text{sin}\left(\theta_{2}/2\right),\;c_{2}\equiv \text{cos}\left(\theta_{2}/2\right)\\ s_{3} & \equiv \text{sin}\left(\theta_{3}/2\right),\;c_{3}\equiv \text{cos}\left(\theta_{3}/2\right) \end{array}$$ (14)

Where *s*_2_ ≥ 0, *c*_2_ ≥ 0. Taking ***e*** and ***e***′ to be orthogonal unit vectors (***e*** ⋅ ***e***′ = 0), there is a third unit vector which is orthogonal to the other two such that:

$$\begin{array}{c} e^{\prime \prime }\equiv \varepsilon \;e\times e^{\prime } \end{array}$$ (15)

Where *ε* = (***e*** × ***e***′) ⋅ ***e***′′ = ±1. Together, ***e***, ***e***′ and ***e***′′ form an orthonormal basis. We also define:

$$\begin{array}{c} \theta_{+}=\frac{\theta_{1}+\theta_{3}}{2}\\ \theta_{-}=\frac{\theta_{1}-\theta_{3}}{2} \end{array}$$ (16)

And:

$$\begin{array}{cc} s_{+} & \equiv \text{sin}(\theta_{+})=s_{1}c_{3}+c_{1}s_{3}\\ s_{-} & \equiv \text{sin}(\theta_{-})=s_{1}c_{3}-c_{1}s_{3}\\ c_{+} & \equiv \text{cos}(\theta_{+})=s_{1}s_{3}-c_{1}c_{3}\\ c_{-} & \equiv \text{cos}(\theta_{-})=s_{1}s_{3}+c_{1}c_{3} \end{array}$$ (17)

Analyzing Eq 13:

$$\begin{array}{cc} q & =[\begin{array}{c} c_{3}\\ s_{3}e \end{array}]⊙[\begin{array}{c} c_{2}\\ s_{2}e^{\prime } \end{array}]⊙[\begin{array}{c} c_{1}\\ s_{1}e \end{array}]\\ & =c_{2}[\begin{array}{c} c_{3}\\ s_{3}e \end{array}]⊙[\begin{array}{c} c_{1}\\ s_{1}e \end{array}]+s_{2}[\begin{array}{c} c_{3}\\ s_{3}e \end{array}]⊙[\begin{array}{c} 0\\ e^{\prime } \end{array}]⊙[\begin{array}{c} c_{1}\\ s_{1}e \end{array}]\\ & =c_{2}[\begin{array}{c} c_{+}\\ s_{+}e \end{array}]+s_{2}[\begin{array}{c} 0\\ c_{-}e^{\prime }+s_{-}e\times e^{\prime } \end{array}] \end{array}$$ (18)

And noting that ***e*** × ***e***′ = *ε* ***e***′′:

$$\begin{array}{c} q=[\begin{array}{c} c_{2}c_{+}\\ c_{2}s_{+}e+s_{2}c_{-}e^{\prime }+s_{2}s_{-}\varepsilon e^{\prime \prime } \end{array}] \end{array}$$ (19)

Defining the following four components:

$$\begin{array}{cc} \left[\begin{array}{c} a\\ b\\ c\\ d \end{array}\right] & \equiv [\begin{array}{c} q_{\text{r}}\\ q\cdot e\\ q\cdot e^{\prime }\\ \varepsilon \;q\cdot e^{\prime \prime } \end{array}] \end{array}$$ (20)

We obtain:

$$\begin{array}{c} \left[\begin{array}{c} a\\ b\\ c\\ d \end{array}]=[\begin{array}{c} c_{2}c_{+}\\ c_{2}s_{+}\\ s_{2}c_{-}\\ s_{2}s_{-} \end{array}\right] \end{array}$$ (21)

Alternatively, we can see that ${\left[\begin{array}{ccc} b & c & d \end{array}\right]}^{T}$ is simply a permutation of the components of ***q***:

$$\begin{array}{c} {}[\begin{array}{c} b\\ c\\ d \end{array}]={\left[\begin{array}{ccc} e & e^{\prime } & e\times e^{\prime } \end{array}\right]}^{T}q \end{array}$$ (22)

#### 3.1.1 Extraction of angles

Using complex numbers, we can define:

$$\begin{array}{c} z_{+}\equiv a+ib=c_{2}\left(c_{+}+is_{+}\right)\\ z_{-}\equiv c+id=s_{2}\left(c_{-}+is_{-}\right) \end{array}$$ (23)

Since *c*_2_, *s*_2_ ≥ 0, we know that |*z*_+_| = *c*_2_, arg(*z*_+_) = *θ*_+_, |*z*_−_| = *s*_2_ and arg(*z*_−_) = *θ*_−_. We can then rewrite:

$$\begin{array}{c} z_{+}=a+ib=c_{2}\text{exp}(i\theta_{+})\\ z_{-}=c+id=s_{2}\text{exp}(i\theta_{-}) \end{array}$$ (24)

And we know that:

$$\begin{array}{c} \theta_{+}=\frac{\theta_{3}+\theta_{1}}{2}=\text{arg}\left\{a+ib\right\}=\text{atan2}\left(b,a\right)\\ \theta_{-}=\frac{\theta_{3}-\theta_{1}}{2}=\text{arg}\left\{c+id\right\}=\text{atan2}\left(d,c\right) \end{array}$$ (25)

#### 3.1.2 Singularities

There are two different singularities in these expressions. When *θ*_2_ = 0, we have *s*_2_ = 0 and *θ*_−_ is undefined. When *θ*_2_ = *π*, we have *c*_2_ = 0 and *θ*_+_ is undefined. In both cases, one degree of freedom is lost and we can argue that *θ*_1_ (or alternatively, *θ*_3_) loses its geometrical meaning. We can then either set *θ*_1_ to zero, or keep it fixed in its latest value (for example, when updating an estimator, for the sake of continuity). Defining:

$$\begin{array}{c} \theta_{1}\equiv {\hat{\theta }}_{1}\;\text{,}\;\text{if}\;\theta_{2}=0\;\text{or}\;\theta_{2}=\pi \end{array}$$ (26)

Taking ${\hat{\theta }}_{1}$ to be some constant (zero or otherwise), we can calculate:

$$\left\{ \begin{array}{c} \theta_{3}=2\;\text{atan2}\left(b,a\right)-{\hat{\theta }}_{1}\;\text{,}\;\text{when}\;\theta_{2}=0\\ \theta_{3}=2\;\text{atan2}\left(d,c\right)+{\hat{\theta }}_{1}\;\text{,}\;\text{when}\;\theta_{2}=\pi \end{array}\right.$$ (27)

#### 3.1.3 General formula for *θ*_1_ and *θ*_3_ in the absence of singularities

If *θ*_2_ ≠ 0 and *θ*_2_ ≠ *π*/2, multiplying *z*_+_ and *z*_−_ yields:

$$\begin{array}{cc} z_{+}\;z_{-} & =(a+ib)(c+id)\\ & =c_{2}s_{2}\text{exp}(i\frac{\theta_{3}+\theta_{1}+\theta_{3}-\theta_{1}}{2})\\ & =c_{2}s_{2}\text{exp}\left(i\theta_{3}\right) \end{array}$$ (28)

On similar lines, multiplying *z*_+_ and the conjugate of *z*_−_ yields:

$$\begin{array}{cc} z_{+}\;z_{-}^{*} & =(a+ib)(c-id)\\ & =c_{2}s_{2}\text{exp}\left(i\theta_{1}\right) \end{array}$$ (29)

The angles can then be obtained using:

$$\begin{array}{c} \theta_{1}=\text{arg}\left(z_{+}\;z_{-}^{*}\right)=\text{arg}\left(\left(a+ib\right)\left(c-id\right)\right)\\ \theta_{3}=\text{arg}\left(z_{+}\;z_{-}\right)=\text{arg}\left(\left(a+ib\right)\left(c+id\right)\right) \end{array}$$ (30)

Or, more simply, from Eq 25:

$$\begin{array}{cc} \theta_{1} & =\text{arg}(a+ib)-\text{arg}(c+id)\\ \theta_{3} & =\text{arg}(a+ib)+\text{arg}(c+id) \end{array}$$ (31)

Or:

$$\begin{array}{c} \theta_{1}=\theta_{+}-\theta_{-}\\ \theta_{3}=\theta_{+}+\theta_{-} \end{array}$$ (32)

It is worth noting that Eq 32 requires fewer operations than Eq 30: only 2 calls to atan2, one addition and one subtraction, but a final wrapping step may be required in order to either keep the angles either in (−*π*, *π*] or [0, 2*π*).

#### 3.1.4 General formulas for calculating *θ*_2_

From Eq 24, we know that:

$$\begin{array}{c} c_{2}=\text{cos}\left(\theta_{2}/2\right)=\left|z_{+}\right|=\sqrt{a^{2}+b^{2}}\\ s_{2}=\text{sin}\left(\theta_{2}/2\right)=\left|z_{-}\right|=\sqrt{c^{2}+d^{2}} \end{array}$$ (33)

And we can use any of the following equivalent formulas obtained directly from the definition:

$$\begin{array}{c} \theta_{2}=2\;\text{asin}(\sqrt{\frac{c^{2}+d^{2}}{n^{2}}})=2\;\text{acos}(\sqrt{\frac{a^{2}+b^{2}}{n^{2}}})=2\;\text{atan}(\sqrt{\frac{c^{2}+d^{2}}{a^{2}+b^{2}}}) \end{array}$$ (34)

Where the factor *n*^2^ = *a*^2^ + *b*^2^ + *c*^2^ + *d*^2^ = |*q*|^2^ can be ignored if the quaternion is already normalized. Using the properties of inverse trigonometric functions, we can also find the following formula, which avoids the need for a square root:

$$\begin{array}{c} \theta_{2}=\text{acos}(2\;\frac{a^{2}+b^{2}}{n^{2}}-1) \end{array}$$ (35)

### 3.2 Case 2: Tait-Bryan angles

We now define:

$$\begin{array}{cc} q & =[\begin{array}{c} c_{3}\\ s_{3}e^{\prime \prime } \end{array}]⊙[\begin{array}{c} c_{2}\\ s_{2}e^{\prime } \end{array}]⊙[\begin{array}{c} c_{1}\\ s_{1}e \end{array}] \end{array}$$ (36)

Where −*π*/2 < *ϕ*_2_ < *π*/2. Again assuming that ***e***, ***e***′ and ***e***′′ are orthogonal unit vectors and ***e***′′ = *ε* ***e*** × ***e***′, where *ε* = (***e*** × ***e***′) ⋅ ***e***′′ = ±1, we define:

$$\begin{array}{c} \lambda \equiv [\begin{array}{c} \text{cos}(\pi /4)\\ \text{sin}\left(\pi /4\right)e^{\prime } \end{array}]=\frac{1}{\sqrt{2}}[\begin{array}{c} 1\\ e^{\prime } \end{array}] \end{array}$$ (37)

We note that:

$$\begin{array}{cc} \lambda^{*}⊙[\begin{array}{c} c_{3}\\ s_{3}\varepsilon e \end{array}]⊙\lambda & =[\begin{array}{c} c_{3}\\ s_{3}\varepsilon e\times e^{\prime } \end{array}]\\ & =[\begin{array}{c} c_{3}\\ s_{3}e^{\prime \prime } \end{array}] \end{array}$$ (38)

Which gives:

$$\begin{array}{c} q=[\begin{array}{c} c_{3}\\ s_{3}e^{\prime \prime } \end{array}]⊙[\begin{array}{c} c_{2}\\ s_{2}e^{\prime } \end{array}]⊙[\begin{array}{c} c_{1}\\ s_{1}e \end{array}]\\ q=\lambda^{*}⊙[\begin{array}{c} c_{3}\\ s_{3}\varepsilon e \end{array}]⊙\lambda ⊙[\begin{array}{c} c_{2}\\ s_{2}e^{\prime } \end{array}]⊙[\begin{array}{c} c_{1}\\ s_{1}e \end{array}]\\ q^{\prime }=[\begin{array}{c} c_{3}^{\prime }\\ s_{3}^{\prime }e \end{array}]⊙[\begin{array}{c} c_{2}^{\prime }\\ s_{2}^{\prime }e^{\prime } \end{array}]⊙[\begin{array}{c} c_{1}\\ s_{1}e \end{array}] \end{array}$$ (39)

Where:

$$\begin{array}{cc} s_{2}^{\prime } & \equiv \text{sin}\theta_{2}^{\prime }/2\;\left(\geq 0\right)\\ c_{2}^{\prime } & \equiv \text{cos}\theta_{2}^{\prime }/2\;\left(\geq 0\right)\\ s_{3}^{\prime } & \equiv \text{sin}\theta_{3}^{\prime }/2\\ c_{3}^{\prime } & \equiv \text{cos}\theta_{3}^{\prime }/2 \end{array}$$ (40)

Where $\theta_{2}^{\prime }=\theta_{2}+\pi /2$ and $\theta_{3}^{\prime }=\varepsilon \theta_{3}$, and:

$$\begin{array}{cc} q^{\prime } & \equiv \lambda ⊙q\\ & =\frac{1}{\sqrt{2}}[\begin{array}{c} 1\\ e^{\prime } \end{array}]⊙[\begin{array}{c} a\\ be+ce^{\prime }+de^{\prime \prime } \end{array}]\\ & =\frac{1}{\sqrt{2}}[\begin{array}{c} a-c\\ \left(b+d\right)e+\left(c+a\right)e^{\prime }+\left(d-b\right)e^{\prime \prime } \end{array}] \end{array}$$ (41)

#### 3.2.1 General formula

Using Eq 41, we can define:

$$\begin{array}{cc} \left[\begin{array}{c} a^{\prime }\\ b^{\prime }\\ c^{\prime }\\ d^{\prime } \end{array}\right] & =\frac{1}{\sqrt{2}}[\begin{array}{c} a-c\\ b+d\\ c+a\\ d-b \end{array}] \end{array}$$ (42)

And then calculate *θ*_1_, $\theta_{2}^{\prime }$ and $\theta_{3}^{\prime }$ using the formulas obtained in the proper case. Using the acos formula for *θ*_2_, we have:

$$\begin{array}{cc} \theta_{2} & =\theta_{2}^{\prime }-\pi /2\\ & =\;\text{acos}(2\;\frac{a^{\prime 2}+b^{\prime 2}}{n^{\prime 2}}-1)-\pi /2 \end{array}$$ (43)

Which results in singularities when $\theta_{2}^{\prime }=0$ or $\theta_{2}^{\prime }=\pi$, which is equivalent to *θ*_2_ = −*π*/2 or *θ*_2_ = *π*/2, as was to be expected. In addition, we know that when no singularities are present:

$$\begin{array}{cc} \theta_{1} & =\;\text{atan2}\left(b^{\prime },a^{\prime }\right)-\;\text{atan2}\left(d^{\prime },c^{\prime }\right)\\ \theta_{3} & =\varepsilon (\;\text{atan2}(b^{\prime },a^{\prime })+\;\text{atan2}(d^{\prime },c^{\prime })) \end{array}$$ (44)

### 3.3 Example of a proper sequence: The sequence ZYZ

If we decide to use the sequence ZYZ, then ***e*** = ***e***_*z*_, ***e***′ = ***e***_*y*_ and ***e***′′ = ***e***_*z*_ × ***e***_*y*_ = −***e***_*x*_. This leads to:

$$\begin{array}{cc} \left[\begin{array}{c} a\\ b\\ c\\ d \end{array}\right] & =[\begin{array}{c} q_{\text{r}}\\ q_{\text{z}}\\ q_{\text{y}}\\ -q_{\text{x}} \end{array}] \end{array}$$ (45)

And the general formulas for *θ*_1_, *θ*_2_ and *θ*_3_ (when no singularities are present, and assuming *q* to have been normalized) are:

$$\begin{array}{cc} \theta_{1} & =\;\text{atan2}\left(q_{\text{z}},q_{\text{r}}\right)-\;\text{atan2}\left(-q_{\text{x}},q_{\text{y}}\right)\\ \theta_{2} & =\;\text{acos}(2\;(q_{\text{r}}^{2}+q_{\text{z}}^{2})-1)\\ \theta_{3} & =\;\text{atan2}\left(q_{\text{z}},q_{\text{r}}\right)+\;\text{atan2}\left(-q_{\text{x}},q_{\text{y}}\right) \end{array}$$ (46)

### 3.4 Second example: The sequence XYZ

Using the sequence XYZ, equivalent to the common aeronautical angles, then ***e*** = ***e***_*x*_, ***e***′ = ***e***_*y*_ and ***e***′′ = ***e***_*z*_. This leads to:

$$\begin{array}{cc} \left[\begin{array}{c} a^{\prime }\\ b^{\prime }\\ c^{\prime }\\ d^{\prime } \end{array}\right] & =\frac{1}{\sqrt{2}}[\begin{array}{c} q_{\text{r}}-q_{\text{y}}\\ q_{\text{x}}+q_{\text{z}}\\ q_{\text{y}}+q_{\text{r}}\\ q_{\text{z}}-q_{\text{x}} \end{array}] \end{array}$$ (47)

And the general formulas for *θ*_1_, *θ*_2_ and *θ*_3_ are:

$$\begin{array}{cc} \theta_{1} & =\;\text{atan2}\left(q_{\text{x}}+q_{\text{z}},q_{\text{r}}-q_{\text{y}}\right)-\;\text{atan2}\left(q_{\text{z}}-q_{\text{x}},q_{\text{y}}+q_{\text{r}}\right)\\ \theta_{2} & =\;\text{acos}({\left(q_{\text{r}}-q_{\text{y}}\right)}^{2}+{\left(q_{\text{x}}+q_{\text{z}}\right)}^{2}-1)-\pi /2\\ \theta_{3} & =\;\text{atan2}\left(q_{\text{x}}+q_{\text{z}},q_{\text{r}}-q_{\text{y}}\right)+\;\text{atan2}\left(q_{\text{z}}-q_{\text{x}},q_{\text{y}}+q_{\text{r}}\right) \end{array}$$ (48)

## 4 Complete algorithm

Algorithm 1, presented in this section, implements the conversion method from this work. Assuming that our inputs are $q\in R^{4}$, the rotation quaternion and *i*, *j* and k∈N, an array of integers defining the sequence of angles (for example, [*i*, *j*, *k*] = [323] is equivalent to the sequence *ZYZ*). A Python implementation can be found on [8].

**Algorithm 1**: Complete implementation of conversion between a rotation quaternion and Euler angles in any sequence, setting *θ*_1_ = 0 in case of singularity.

**Input**: $q\in R^{4}$, and *i*, *j*, *k* ∈ {1, 2, 3}, where *i* ≠ *j*, *j* ≠ *k*

**Output**: *θ*_1_, *θ*_2_, *θ*_3_

**if** *i* == *k* **then**

 *not_proper* ← False

 *k* ← 6 − *i* − *j* // because *i* + *j* + *k* = 1 + 2 + 3 = 6

**else**

 *not_proper* ← True

**end**

*ε* ← (*i* − *j*) × (*j* − *k*) × (*k* − *i*)/2 // equivalent to the Levi-Civita symbol

**if** *not_proper* **then**

 *a* ← *q*[0] − *q*[*j*]

 *b* ← *q*[*i*] + *q*[*k*] × *ε*

 *c* ← *q*[*j*] + *q*[0]

 *d* ← *q*[*k*] × *ε* − *q*[*i*]

**else**

 *a* ← *q*[0]

 *b* ← *q*[*i*]

 *c* ← *q*[*j*]

 *d* ← *q*[*k*] × *ε*

**end**

$\theta_{2}\leftarrow \;\text{acos}[2\;(\frac{a^{2}+b^{2}}{a^{2}+b^{2}+c^{2}+d^{2}})-1]$

*θ*^+^ ← atan2(*b*, *a*)

*θ*^−^ ← atan2(*d*, *c*)

**switch** *value of θ*_2_ **do**

 **case** 0 **do**

  *θ*_1_ ← 0 // For simplicity, we are setting ${\hat{\theta }}_{1}=0$

  *θ*_3_ ← 2 × *θ*^+^ − *θ*_1_

 **case** *π*/2 **do**

  *θ*_1_ ← 0

  *θ*_3_ ← 2 × *θ*^−^ + *θ*_1_

 **otherwise do**

  *θ*_1_ ← *θ*^+^ − *θ*^−^

  *θ*_3_ ← *θ*^+^ + *θ*^−^

 **end**

**end**

**if** *not_proper* **then**

 *θ*_3_ ← *ε* × *θ*_3_

 *θ*_2_ ← *θ*_2_ − *π*/2

**end**

*θ*_1_, *θ*_3_ ← (*θ*_1_, *θ*_3_) // “wrap” assures *θ*_1_, *θ*_3_ ∈ (−*π*, *π*] or *θ*_1_, *θ*_3_ ∈ [0, 2*π*)

Many operations are required to convert a quaternion into a rotation matrix. Using the homogeneous formula from Eq 11, for example, if special care is taken in order not to repeat any operations, we have to perform at least 4^2^ = 16 floating point multiplications (all the possible products between two different components of the quaternion, plus all the squares of each component), 4 × 3 = 12 multiplications by 2 and 3 × 3 + 6 = 15 additions/subtractions. This conversion step alone is more than enough to make an algorithm based on [5] much slower than the proposed method. In addition, multiple matrix multiplications also have to be computed. By comparison, our algorithm replaces all the conversions and matrix multiplications by a simple permutation of the quaternion elements and in the case of Tait-Bryan angles, only 5 additional additions/subtractions and possibly a sign change are required.

## 5 Results

In this section, a performance comparison between our method and the Shuster method is presented. We adapted the SciPy library in order to compile the algorithm as described in Section 4. A real data set comprising the orientation of a spinning object with 3284 data points was used to compare the efficiency of the two algorithms. The full implementation and data set can be downloaded from [8]. First we noted that both methods give the same results: adding the absolute value of the differences between the two methods in a whole data set gives an error of the order of 10^−12^. The execution times required in our tests for each sequence (and their ratios) are presented in the Table 1. From this test, it can be clearly seen that the method presented here is about 30 times faster.

**Table 1:** Comparison of execution times between the two methods. The Python module *timeit* was used to check the execution time required to convert the whole data set 500 times on an Intel^®^ Core^™^ i3-4030U CPU with a 1.90GHz clock speed.

seq	new method	[5] implemented in [6]	ratio
ZYZ	0.487 s	13.770 s	28.261
ZXZ	0.384 s	13.361 s	34.805
XYX	0.382 s	13.381 s	34.998
XZX	0.414 s	13.187 s	31.832
YXY	0.359 s	13.029 s	36.262
YZY	0.375 s	13.078 s	34.884
ZYX	0.371 s	13.152 s	35.408
ZXY	0.364 s	13.124 s	36.048
XYZ	0.373 s	13.170 s	35.291
XZY	0.385 s	13.157 s	34.213
YXZ	0.365 s	13.087 s	35.838
YZX	0.425 s	13.122 s	30.844

## 6 Conclusion

The Euler angles are still a useful intuitive 3D orientation parametrization. A fast method of conversion to/from any other set of parameters can therefore be of great interest for displaying or analyzing data, for instance. In this study, we therefore developed a general formula for this conversion which is concise, easy to implement and easy to debug. In addition, the fact that our method is about 30 times faster than the method proposed by [5], which required an intermediate conversion into rotation matrices, we believe that our proposed method can be of great interest. This faster execution time also makes this method suitable for use in embedded real time applications such as inertial measurement units (IMUs). We propose that this method could be adopted as the new standard method for converting quaternions into Euler angles, and we are now planning to contributing to several scientific libraries accordingly. Moreover, a possible further development is to generalize this formula for the Davenport angles [9], a generalization of the Euler angles in which any set of distinct non-orthogonal axes are used.
